# Impurities in London Water

**Published:** 1899-12-30

**Authors:** 


### Impurities in London Water.

In his annual report as medical officer of health for the parish of St. George, Hanover Square, Professor Corfield (who is also consulting sanitary adviser to her Majesty's Office of Works) reprints an interesting correspondence between himself on the one side and Sir William Crookes and Professor Dewar on the other, in regard to the relation between organic impurity in the filtered Thames water and the prevalence of enteric fever. We cannot but feel that Professor Corfield comes off the victor. His accusation against the London water is founded on the fact that there had been more cases of enteric fever in his parish during November and December than during the preceding three months. "November, December, and January are not the months for the 'seasonal prevalence' of this disease, and therefore some other cause for the excess of it must be sought for, and it may," he says, "be something more than a coincidence that in those very months London is supplied with water which, according to Sir Edward Frankland's report, contains 'an excess of organic matter.'"

Now it is clearly no answer to this to say: "We say deliberately that after mature consideration we know of no example of a water-borne disease having originated from a river supply where proper precautions are taken for adequate storage such as exist in the case of the London water supply." This is begging the very question at issue.

So long as it remains the fact that the water after filtration varies, both in organic impurity and in microbic impurity, with the condition of the water before filtration, we must hesitate to admit that such filtration is an efficient protection against the passage of disease germs. Taking the admissible number of microbes of all kinds which may be allowed in the filtered water at 100 per cubic centimetre, what are we to say to a filtered water which, according to the examinations made by the late Sir Edward Frankland varied in the number of microbes it contained at different times, from 16,000 per cubic centimetre to none at all. It is the extreme variation in the effluent at different times that shakes one's faith in the character of the filtration. It is nothing to us to know that sometimes the water is very good, if the tumblerful which we hold at our lips contains an enormous number of mixed microbes.

The whole question is far too serious to be met by *ad captandum* statements to the effect that the bacteriological purification which the water has undergone is such as to remove "the suspicion of the possibility of its conveying any waterborne disease." The fact unfortunately remains that it is still under very grave suspicion, and that this suspicion has been expressed by people well versed on the subject, and occupying responsible official positions.

If the companies wish to show that their arrangements are an efficient protection against the diseases caused by micro-organisms, let them show that they can maintain an effluent from their filters which is equally free from micro-organisms whatever the character of the water from which it is drawn. This they cannot at present do, and it seems to us that when the analysts employed by the water companies persist in speaking of "adequate storage and effective filtration" as being "such as exist in the case of the London water supply," such happy contentment with the present condition of affairs does not augur well for improvement in the future. This is the bad part of the business.

If we refer to the last report of the Local Government Board we find the following remarks by Major-General Scott, water examiner under the Metropolis Water Act, 1871: "It is necessary for the proper treatment of the water that storage reservoirs, capable of containing water sufficient for 30 days' supply, should be provided by each company."

On another page of the same report is a table giving the storage and filtering areas available in the case of each company, from which it appears that only in the case of one company, namely, the East London, is the above-mentioned amount of storage provided, the Lambeth Company only having 5·1 days' supply, the New River 4·5, and the Grand Junction Company the absurdly inefficient amount of only 3·2 days' supply.

